# Identification of warning signs for prevention of in-hospital cardiorespiratory arrest^*^

**DOI:** 10.1590/1518-8345.2853.3072

**Published:** 2019-01-31

**Authors:** Beatriz Tessorolo Souza, Maria Carolina Barbosa Teixeira Lopes, Meiry Fernanda Pinto Okuno, Ruth Ester Assayag Batista, Aécio Flávio Teixeira de Góis, Cássia Regina Vancini Campanharo

**Affiliations:** 1 Universidade Federal de São Paulo, Escola Paulista de Enfermagem, São Paulo, SP, Brazil.; 2 Universidade Federal de São Paulo, Escola Paulista de Medicina, São Paulo, SP, Brazil.

**Keywords:** Emergency Nursing, Cardiopulmonary Arrest, Vital Signs, Hospital Care, Secondary Prevention, Quantitative Research, Enfermagem em Urgência e Emergência, Parada Cardiorrespiratória, Sinais Vitais, Assistência Hospitalar, Prevenção Secundária, Pesquisa Quantitativa, Enfermería de Emergencia, Paro Cardíaco, Signos Vitales, Atención Hospitalaria, Prevención Secundaria, Investigación Cuantitativa

## Abstract

**Objective:**

to identify the occurrence of warning signs and changes in vital signs in individuals who experienced in-hospital cardiorespiratory arrest and correlate them with the occurrence of this event.

**Method:**

this is a retrospective, analytical and quantitative study that included 218 medical records of patients who suffered in-hospital cardiorespiratory arrest and identified warning signs and alterations in vital signs. Mean, standard deviation, median, minimum and maximum values were calculated for the continuous variables, and frequency and percentage for the categorical variables. We compared the age and occurrence of cardiorespiratory arrest with the occurrence of warning signs using the Chi-Square Test and the Mann Whitney non-parametric test (p-value < 0.05).

**Results:**

62.1% of the patients presented signs and symptoms of shock, 44.9% of neurological alteration, 40.4% of malaise, 15.2% presented signs suggestive of acute coronary syndrome, and 25.9% presented mental confusion. In the last measurement of vital signs before cardiorespiratory arrest, the majority of patients had altered abnormal (32.6%) and severely abnormal (23.9%) heart rate, and abnormal (37.1%) and severely abnormal (27.0%) respiratory rate.

**Conclusion:**

the warning signs identified were: shock, neurological signs, malaise and acute coronary syndrome. The prevalent changes in vital signs were: heart rate, respiratory rate and O_2_ saturation. Patients with severely abnormal systolic blood pressure were not discharged and those with abnormal respiratory rate did not survive 6 months after cardiorespiratory arrest.

## RESUMO

**Objetivo:**

identificar ocorrência dos sinais de alerta e alterações nos sinais vitais em indivíduos com parada cardiorrespiratória intra-hospitalar e correlacioná-los à ocorrência desse evento.

**Método:**

estudo retrospectivo, analítico e quantitativo que incluiu 218 prontuários de pacientes que sofreram parada cardiorrespiratória intra-hospitalar e identificados sinais de alerta e alterações nos sinais vitais. Para variáveis contínuas, calculou-se média, desvio padrão, mediana, mínimo e máximo; para as categóricas, frequência e percentual. Comparou-se a idade e ocorrência de parada cardiorrespiratória com ocorrência de sinais de alerta pelo Teste Qui-Quadrado e Teste não paramétrico de MannWhitney (p-valor<0,05).

**Resultados:**

62,1% dos pacientes apresentaram sinais e sintomas de choque, 44,9% neurológicos, 40,4% mal-estar, 15,2% sugestivos de síndrome coronariana aguda e 25,9% confusão mental. Na última mensuração dos sinais vitais antes da parada cardiorrespiratória, a maioria apresentou frequência cardíaca alterada, anormal (32,6%) e severamente anormal (23,9%), frequência respiratória anormal (37,1%) e severamente anormal (27,0%).

**Conclusão:**

identificou-se como sinais de alerta: sinais de choque, neurológicos, mal-estar e síndrome coronariana aguda. Alterações nos sinais vitais prevalentes foram: frequência cardíaca, respiratória e saturação de O2. Pacientes com pressão arterial sistólica severamente anormal não receberam alta e aqueles com frequência respiratória anormal não sobreviveram em 6 meses após a parada cardiorrespiratória.

## RESUMEN

**Objetivo:**

identificar la presencia de señales de alerta y alteraciones en los signos vitales en individuos con parada cardiorrespiratoria intrahospitalaria y correlacionarlos a la ocurrencia de ese evento.

**Método:**

estudio retrospectivo, analítico y cuantitativo que incluyó 218 prontuarios de pacientes que sufrieron paro cardiorrespiratorio intrahospitalario e identificados signos de alerta y alteraciones en los signos vitales. Para variables continuas, se calculó la media, desviación estándar, mediana, mínima y máxima; para las categóricas, se calculó la frecuencia y el porcentaje. Se comparó la edad y ocurrencia de parada cardiorrespiratoria con ocurrencia de señales de alerta por el Test Chi-cuadrado y Test no paramétrico de MannWhitney (p-valor <0,05).

**Resultados:**

62,1% de los pacientes presentaron signos y síntomas de shock, 44,9% neurológicos, 40,4% malestar, 15,2% sugestivos de síndrome coronario agudo y 25,9% confusión mental. En la última medición de los signos vitales antes de la parada cardiorrespiratoria, la mayoría presentó frecuencia cardiaca alterada, anormal (32,6%) y severamente anormal (23,9%), frecuencia respiratoria anormal (37,1%) y severamente anormal (27,0%).

**Conclusión:**

como signos de alerta se identificaron: signos de shock, neurológicos, malestar y síndrome coronario agudo. Los cambios en los signos vitales prevalentes fueron: frecuencia cardíaca, respiratoria y saturación de O_2_. Los pacientes con presión arterial sistólica severamente anormal no recibieron alta y aquellos con frecuencia respiratoria anormal no sobrevivieron en 6 meses después de la parada cardiorrespiratoria.

## Introduction

The nursing team is often the first to identify clinical changes in patients. These changes can be easily detected by monitoring vital signs (VS) and careful observation of the patient’s facial expressions and neuro-emotional behavior. The identification of changes in deviant values is accompanied by an increased risk of adverse clinical events such as cardiorespiratory arrest (CRA). Early identification of abnormalities offers the opportunity for timely intervention and increased survival with better quality of life of patients^1^.

CRA is characterized by sudden interruption of the heart rate, respiratory movements and immediate loss of consciousness, leading to irreversible brain damage and death if adequate measures to stabilize the patient are not taken immediately^2^.

Annually, more than 200,000 adults undergo in-hospital CRA in the United States^3^ ^-^ ^4^, and many of these events could have been prevented^5^ ^-^ ^6^ through early identification of signs and initiation of appropriate therapy^5^.

CRA is rarely a sudden event. It is the result of progressive deterioration of respiratory and circulatory function^7^. CRA in hospitalized patients is often preceded by signs of clinical worsening. Early detection and intervention in situations of clinical instability is an opportunity to prevent CRA in these patients and increase the safety of hospitalized patients^8^.

Studies have demonstrated the relationship between abnormalities in routine measures of VS and poor outcomes, including death and in-hospital CRA^9^ ^-^ ^10^. An American study found a high prevalence of abnormal VS preceding CRA. Patients with three abnormal VS had 20% higher mortality than those without alterations, showing the direct relationship between these changes and the increase in in-hospital mortality rate^11^. A study in Japan implemented an Early Warning Score (EWS) system in which each change in a vital signal (systolic blood pressure, heart rate, respiratory rate, temperature, level of consciousness) was given a value of 0-3; the total score corresponded to the sum of these values. Scores greater than or equal to 7 corresponded to “danger zone”, that is, a greater possibility of acute deterioration. Patients in the “danger zone” received early interventions. After the implantation of the EWS, the rate of in-hospital CRA per 1000 admissions decreased from 5.21 to 2.05^12^.

The measurement of VS, which is usually the responsibility of the nursing team, is a routine and extremely important hospital activity, because it determines the health status of the individuals, the evolution of the clinical picture, and can predict clinical deterioration^1^.

The first link in the chain of survival for in-hospital CRA is surveillance of patients and identification of warning signs. The literature cites changes in VS as risk factors for CRA. The performance of the nursing team in the periodic verification of VS is relevant in this scenario, to the identification of early changes that may precede CRA and other cardiovascular emergencies, thus increasing patient safety.

The objective of this study was to identify the occurrence of warning signs and changes in VS in individuals who presented in-hospital CRA and correlate the presence of warning signs and changes in VS with the occurrence of CRA.

## Method

This is a retrospective, analytical and quantitative study performed at the Emergency Unit of the Hospital of São Paulo (HSP). The HSP is a large, high complexity, university hospital that offers multiprofessional health care in ambulatory, hospitalization and urgency and emergency modalities^13^.

Data collection was performed from October 2016 to January 2017, retrospectively, through the analysis of medical records of patients. A total of 218 medical records of patients who experienced CRA in the Clinical Emergency sector from January 2011 to February 2012 were included in the study. Patients who had a CRA who had out-of-hospital cardiac arrest were excluded. Variables of patients were collected using an instrument created by the researcher, including: gender, age, skin color, neurological status before CRA, and presence of previous CRA. The variables related to the CRA were: initial rhythm, time intervals between cardiopulmonary resuscitation (CPR) attempts, defibrillation, airway clearance, and epinephrine and CRA duration. Warning signs such as neurological changes (lowering of consciousness level, convulsion, hemiparesis, deviation of rhyme and lip movement, dysarthria, slurred speech and/or speech different from the usual pattern), symptoms of acute coronary syndrome (ACS) (chest pain, skin color and humidity changes, and decreased temperature at the extremities - pale, grayish, moist or cyanotic extremities) and changes in vital signs were recorded in the 24 hours preceding CRA. We also investigated whether there was a return of spontaneous circulation and survival in the first 24 hours, at discharge, and six months and one year after the CRA. In the case of patients who died, the cause of death was investigated. 

Descriptive and inferential analyses of data were carried out in the SPSS software (Statistical Package for the Social Sciences, version 11.5 for Windows). Statistical analyses included the calculation of mean, median, minimum and maximum values of the continuous variables. In the case of the categorical variables, frequencies and percentages were calculated. The QUI-Square test was used to calculate the occurrence of death with variables of interest, and, when necessary, the Fisher’s exact test or the Likelihood Ratio Test were used. Cox Regression was used to identify factors related to patient survival. A significance level of 5% (p-value < 0.05) was adopted. The Fisher’s exact test was used to compare the evolution of Cerebral Performance Category (CPC) with variables of interest (categorical). The Mann-Whitney test was used to compare the occurrence of death with variables of interest (continuous); a significance level of 5% (p-value < 0.05) was adopted. The results were presented through tables and graphs. For statistical analysis of VS, the following values were considered abnormal: heart rate (HR) ≤ 60 or ≥ 100bpm, respiratory rate (RR) ≤ 10 or > 20 rpm and systolic blood pressure (SBP) ≤ 90mmHg. A subgroup of severely abnormal VS was also considered: HR ≤ 50 or ≥ 130bpm, RR ≤ 8 or ≥ 30rpm and SBP ≤ 80 mmHg^11^. 

This study is part of a doctoral thesis approved by the Research Ethics Committee of the Federal University of São Paulo (protocol 0030/2011). Considering that this study is observational and that data collection was done by means of medical records, not causing any type of interference in the sector or in patient care, the study was exempt from informed consent term.

## Results

The mean age of the study population was 66.8 years, with 52.3% of males and 47.7% of females. Regarding color, 71.1% declared to be white, 15.1% yellow, 10.6% black and 3.2% brown. Most were independent in activities of daily living (53.9%) and had not had a previous CRA (97.7%).

According to Table 1, the most frequent initial rhythm of CRA was pulseless electrical activity (57.4%), and the mean time between collapse and onset of CPR maneuvers was 0.8 minutes. The mean time between onset of CPR and the 1st shock was 9.4 minutes; between onset of CPR and airway clearance was 5.5 minutes; and between onset of CPR and administration of the 1st dose of epinephrine was 1.7 minutes. The total duration of CPR was 16.1 minutes, on average.

**Table 1:** Initial cardiac arrest rhythm and time intervals during care in the studied population. São Paulo, SP, Brazil, 2017

**Clinical variables**	**n (%)**
**Initial rhythm**	
**Ventricular fibrillation**	**9 (4.2)**
**Pulseless ventricular tachycardia**	**6 (2.8)**
**Pulseless electrical activity**	**124 (57.4)**
**Asystolia**	**77 (35.6)**
**Total patients**	**216**
Time collapse - onset of CPR*	
**Mean (SD)**	**0.8 (2.8)**
**Median (Minimum - Maximum)**	**0 (0-21)**
**Total patients**	**157**
Time onset of CPR* - 1st shock	
**Mean (SD)**	**9.4 (12.2)**
**Median (Minimum - Maximum)**	**4 (0-55)**
**Total patients**	**33**
Time onset of CPR* - UAW clearance ^**†**^	
**Mean (SD)**	**5.5 (6.0)**
**Median (Minimum - Maximum)**	**4 (0-35)**
**Total patients**	**92**
Time onset of CPR* - 1st epinephrine	
**Mean (SD)**	**1.7 (2.5)**
**Median (Minimum - Maximum)**	**1 (0-17)**
**Total patients**	**150**
Time onset of CPR* - end of CPR*	
**Mean (SD)**	**16.1 (13.0)**
**Median (Minimum - Maximum)**	**13 (2-76)**
**Total patients**	**165**

*CPR - Cardiopulmonary Resuscitation; ^†^UAW - upper airways;

Concerning the warning signs for the occurrence of CRA (n = 198), 62.1% of the patients presented signs and symptoms of shock; 44.9% presented neurological signs; 40.4% presented malaise; and 15.2% had signs and symptoms suggestive of acute coronary syndrome.

The mean values of the following variables in the 24 hours preceding CRA in the studied population were: respiratory rate: 25.5 bpm; systolic blood pressure: 98.3 mmhg; diastolic blood pressure: 60.0mmhg; mean blood pressure: 60.1mmhg; heart rate: 84.7bpm; temperature: 36.3°C; oxygen saturation: 90.4%; and mental confusion: 25.9% of the patients. These data are shown in Table 2.

**Table 2:** Vital signs, oxygen saturation and level of consciousness in the 24 hours preceding the cardiorespiratory arrest in the studied population. São Paulo, SP, Brazil, 2017

**Clinical variables**	**n (%)**
**Respiratory frequency**	
**Mean (Standard deviation)**	**25.5 (8.7)**
**Median (Minimum-Maximum)**	**24 (8-48)**
**Total patients**	**116**
**Systolic Blood Pressure**	
**Mean (SD)**	**98.3 (33.2)**
**Median (Minimum - Maximum)**	**97.5 (30-200)**
**Total patients**	**180**
**Diastolic Blood Pressure**	
**Mean (SD)**	**60 (22.2)**
**Median (Minimum - Maximum)**	**60 (20-131)**
**Total patients**	**180**
**Mean Blood Pressure**	
**Mean (SD)**	**60.1 (35.8)**
**Median (Minimum - Maximum)**	**63.7 (0-145)**
**Total patients**	**218**
**Heart rate**	
**Mean (SD)**	**84.7 (30.2)**
**Median (Minimum - Maximum)**	**84 (8-148)**
**Total patients**	**184**
**Temperature**	
**Mean (SD)**	**36.3 (1.6)**
**Median (Minimum - Maximum)**	**36 (32-41.6)**
**Total patients**	**117**
**O**_**2**_ **saturation**	
**Mean (SD)**	**90.4 (8.5)**
**Median (Minimum - Maximum)**	**93 (35-100)**
**Total patients**	**144**
**Level of consciousness**	**n (%)**
**Alert**	**23 (20.5)**
**Confuse**	**29 (25.9)**
**Responds to pain**	**7 (6.3)**
**Unconscious**	**21 (18.8)**
**Sedated**	**24 (21.4)**
**Responds to verbal stimulation**	**8 (7.1)**
**Total patients**	**112**

Regarding outcomes, 48.6% had return of spontaneous circulation, with 16.8% surviving the first 24 hours, 6.3% surviving at hospital discharge, 5.3% surviving six months after discharge, and 4.9% surviving one year after the discharge. Regarding the reasons of death, 92 (42.6%) died from infection, 52 (24.1%) from cancer, 39 (18.1%) from cardiovascular diseases, 3 (1.4%) from trauma, 95 (44%) due to other causes.

According to Table 3, most survivors at discharge were independent in daily life activities 6 months and 1 year after the CRA, with CPC 1 or 2.

**Table 3:** Neurological state after cardiorespiratory arrest in the study population. São Paulo, SP, Brazil, 2017

**Clinical variables**	**n (%)**
CPC* at discharge	
**1**	**3 (27.3)**
**2**	**6 (54.5)**
**3**	**1 (9.1)**
**4**	**1 (9.1)**
**Total patients**	**11**
CPC* 6 months after CRA	
**1**	**5 (55.6)**
**2**	**3 (33.3)**
**3**	**1 (11.1%)**
**Total patients**	**9**
CPC* 1 year after CRA	
**1**	**5 (62.5)**
**2**	**3 (37.5)**
**Total patients**	**8**

*CPC - Cerebral Performance Category

Table 4 shows the association of socio-demographic variables, neurological state before CRA, the initial CRA rhythm, and the outcomes with the warning signs presented by the patients in this study.

**Table 4:** Association warning signs with socio-demographic variables, neurological state before cardiorespiratory arrest, initial cardiac arrest rhythm and outcomes. São Paulo, SP, Brazil, 2017

**Variables of interest**	**Neurological signs**		**Total n (%)**	**p-value**
	**Yes n (%)**	**No n (%)**		
**Return of Spontaneous Circulation (N = 198)**				
**Yes**	**56 (62.9)**	**40 (36.7)**	**96 (48.5)**	**0.0002**
**No**	**33 (37.1)**	**69 (63.3)**	**102 (51.5)**	
**Survival in the first 24 hours (N = 198)**				
**Yes**	**20 (22.5)**	**10 (9.2)**	**30 (15.2)**	**0.0094**
**No**	**69 (77.5)**	**99 (90.8)**	**168 (84.8)**	
	Signs of ACS*		**Total n (%)**	**p-value**
	**Yes n (%)**	**No n (%)**		
**CPC** ^**†**^ **before CRA (N = 197)**				
**1**	**12 (33.3)**	**24(66.7)**	**36 (100)**	**0.0023** ^**§**^
**2**	**16 (14.4)**	**95 (85.6)**	**111 (100)**	
**3**	**2 (4.3)**	**45 (95.7)**	**47 (100)**	
**4/5**	**0 (0)**	**4 (100)**	**4 (100)**	
**Initial rhythm (N = 197)**				
**Ventricular fibrillation**	**4 (57.1)**	**3 (42.9)**	**7 (100)**	**0.0296** ^**§**^
**Pulseless ventricular tachycardia**	**1 (20)**	**4 (80)**	**5 (100)**	
**Pulseless electrical activity**	**19 (16.1)**	**99 (8.9)**	**118 (100)**	
**Asystolia**	**6 (9)**	**61 (91)**	**67 (100)**	
**Discharge (N = 196)**				
**Yes**	**4 (13.3)**	**6 (6.3)**	**10 (5.1)**	**0.0486** ^**||**^
**No**	**26 (86.7)**	**160 (96.4)**	**186 (94.9)**	
**Death due to CVD** ^**‡**^ **(N = 196)**				
**Yes**	**10 (33.3)**	**26(16.7)**	**36 (18.4)**	**0.0214**
**No**	**20 (66.7)**	**140 (84.3)**	**160 (81.6)**	
	**Signs of shock**		**Total n (%)**	**p-value**
	**Yes n (%)**	**No n (%)**		
**Age (N = 198)**				
**Mean (SD)**	**69.3 (16.3)**	**63.4 (17)**	**67.1 (16.8)**	**0.0113**
**Median (minimum - maximum)**	**72 (29-101)**	**65 (17-99)**	**70 (17-101)**	
**Survival in the first 24 hours (N = 196)**				
**Yes**	**2 (1.7)**	**8 (10.7)**	**10 (5.1)**	**0.007** ^**||**^
**No**	**119 (98.3)**	**67 (89.3)**	**186 (84.8)**	
**Survival 6 months after CRA (N = 196)**				
**Yes**	**1 (0.8)**	**7 (9.3)**	**8 (4.1)**	**0.0055** ^**§**^
**No**	**120 (99.2)**	**68 (90.7)**	**188 (95.9)**	
**Survival 1 year after CRA (N = 195)**				
**Yes**	**0 (0)**	**7 (9.3)**	**7 (3.6)**	**0.0010** ^**||**^
**No**	**120 (100)**	**68 (90.7)**	**188 (96.4)**	
**Death due to infection (N = 196)**				
**Yes**	**64 (52)**	**27 (37)**	**91 (46.4)**	**0.0412**
**No**	**59 (48)**	**46 (63)**	**105 (53.6)**	

*ACS - Acute Coronary Syndrome; ^†^CPC - Cerebral Performance Category; ^‡^CVD - Cardiovascular Disease; ^§^Likelihood Ratio Test; ^||^Fisher’s Exact Test

Regarding the occurrence of neurological signs, patients who presented these signs had a higher percentage of return of spontaneous circulation (62.9%) and survival in the first 24 hours after the event (22.5%) when compared to those who did not (36.7% and 9.2%, respectively). Among patients who presented signs of Acute Coronary Syndrome (ACS), there was a predominance of patients with CPC 1 (33.3), followed by patients with CPC 2 (14.4%), over patients with CPC 3 and 4/5, which corresponded to 4.3% and 0%, respectively. Patients who presented VF as the initial CRA rhythm had a higher percentage of signs of ACS (57.1%) than patients with other rhythms. Patients with signs of ACS had a higher percentage of discharge (13.3%) and death due to cardiovascular disease (CVD) (33.3%) than those who did not present such signs.

A higher mean age (69.3) and rate of death due to infections (52%) were observed in patients who presented signs of shock when compared to patients who did not presented such signs (63.4 years and 37%, respectively). Patients without signs of shock had a higher percentage of survival, survival in the first 24 hours after CRA (10.7%), 6 months after CRA (9.3%) and 1 year after CRA (9.3%).

Regarding the last measurement of vital signs before CRA, the majority of patients had abnormal and severely abnormal heart rate (32.6% and 23.9%, respectively) and systolic blood pressure (54.4%) within the normality. Regarding respiratory rate, the majority of patients had abnormal and severely abnormal parameters (37.1% and 27.0%, respectively), as presented in Table 5.

**Table 5:** Last measurement of heart rate, respiratory rate and systolic blood pressure before cardiorespiratory arrest, classified as normal/abnormal. São Paulo, SP, Brazil, 2017

Vital signs	n(%)
Heart rate (bpm*)	
Normal	80 (43.5)
Abnormal	60 (32.6)
Severely abnormal	44 (23.9)
Total patients	184
Respiratory Rate (ripm^†^)	
Normal	41 (35.3)
Abnormal	43 (37.1)
Severely abnormal	32 (27.0)
Total patients	116
Systolic Blood Pressure (mmHg^‡^)	
Normal	98 (54.4)
Abnormal	20 (11.1)
Severely abnormal	62 (34.4)
Total patients	180

*bpm - Beats per minute; ^†^ripm - Respiratory incursions per minute; ^‡^mmHg - Millimeters of Mercury;

## Discussion

In the present study, some socio-demographic characteristics of the patients were similar to those reported in the literature, such as higher proportion of white skin and males. The mean age of the patients in this sample was higher than in other national studies conducted in coronary care units and intensive care units. However, international studies on hospitalization and intensive care units (ICUs) found a higher mean age than that found in the present study^14^ ^-^ ^19^. The most frequent initial rhythm of CRA was pulseless electrical activity (57.4%) followed by asystolia (35.6%). In general, these two rhythms are reported as the most common in hospital settings, but asystolia was the most prevalent in an American study evaluating the post-CRA prognosis in patients with chronic diseases aged 18 to more than 80 years^15^ and in another study aimed at determining whether early administration of epinephrine in patients with non-shockable initial CRA rhythm is associated with better neurological prognoses^16^.

In this study, the mean time interval in minutes between collapse and onset of CPR (0.8’), between onset of CPR and defibrillation (9.4’), and between onset of CPR and clearance of UAW (5.5’) were higher than those observed in a prospective study performed in an ICU in Minas Gerais, which presented means of 0.7’; 7.1’; and 4.8’, respectively^18^. This may be related to the fact that this study was performed in an Emergency Service, which may imply a delay in the monitoring of patients presenting CRA, different from the intensive care setting where patients are already monitored. Another aspect that may be related is the fact that this study was retrospective, and the information of interest was taken from hospital records, with possibility of incomplete information.

The mean time interval between onset of CPR and administration of the first dose of epinephrine was 1.7 min in this study, lower than that observed in another study (2.5’). This can be attributed to the fact that the present study was carried out in a university hospital with a large contingent of professionals. Regarding the time between onset and end of CPR, this interval in the studied sample (16.1 ‘) was similar to that of another study performed in an ICU (16.3’)^18^.

The monitoring of VS of patients allows the detection of changes that increase the risk of adverse clinical events such as CRA. A study conducted in the United Kingdom used the National Early Warning Score, a system based on HR, RR, blood pressure, O_2_ saturation, temperature, level of consciousness, and supplemental oxygen use, ranging from 0 to 20, to detect patients at risk of CRA^10^. This study found that mean HR (81 bpm), RR (17 rpm), SBP (126 mmHg), diastolic blood pressure (70 mmHg), temperature (36.7 °C) and O_2_ saturation (96%) were normal^10^, differently from the findings of the present study. Another parameter analyzed in the research was the level of consciousness, in which 91.7% of the patients were alert^10^, different from the patients in the present sample in which the majority had altered level of consciousness (79.5%), being mental confusion the most frequent state (25.9%). This difference can be explained by the fact that all patients in this study progressed to CRA.

Similar studies investigated the prevalence of abnormal vital signs and their association with the risk of cardiorespiratory arrest. Among the VS changes described in the literature associated with the risk of CRA are abnormal HR, abnormal RR and decreased SBP^11^ ^,^ ^20^. In the study, all patients with severely abnormal SBP (≤ 80 mmHg) died. Similar results were observed in another study, where mortality increased as SBP values decreased, and patients with SBP ≤ 80 mmHg had a mortality rate above 90%^11^.

In the current study, the majority of patients (37.1%) presented abnormal respiratory rate, and all patients who presented such change died 6 months after the CRA. However, a study conducted with the objective of examining the association between critical changes in VS and mortality reports that patients with RR < 10 rpm at admission presented 10% mortality^9^, values lower than those found in the present study, and in another study that revealed mortality rates ranging from 80% to 90% for RR values > 20 rpm^11^.

Regarding pre-CRA CPC, the majority of patients in this sample were classified as CPC 2 (53.9%). Other national studies also presented higher percentage of patients with CPC 2 (50%^21^ and 96.7%^19^). The high value of the second study^19^ may be associated with the fact that the study only included patients who were discharged after the event.

The CPC of the patients in this study was reassessed at the moment of hospital discharge and at two other times: six months and one year after the CRA. An improvement of the neurological condition was observed over time. At hospital discharge, most patients had CPC 2, whereas in the following assessments, the percentages of CPC 1 were higher. Similar results were obtained in another national study, with progressive improvement of the neurological condition in these time intervals^21^.

The association of variables in this study with warning signs presented by the patients showed that the occurrence of neurological signs was associated with higher percentages of return to spontaneous circulation. These results may be related to the state of low cerebral perfusion that can precede CRA, evidenced by lowering of consciousness level, occurrence of seizures, and changes in movement, sensitivity and speech, commonly detected by health professionals^22^.

Most patients with signs of ACS presented CPC 1 and 2 before CRA, and as expected, the most frequent CRA rhythm was ventricular fibrillation. Shockable rhythms are common in the first few hours after the onset of ACS symptoms and high-quality immediate CPR and early defibrillation are associated with higher survival and better neurologic outcome in these individuals^22^.

Coronary reperfusion is recommended in patients with suspected or confirmed diagnosis of ACS post-CRA^22^. The highest percentages of patients who showed signs of ACS in this study may be related to the early and specialized care given at the studied hospital, which has a cardiologist in the emergency department and an interventional cardiology sector.

In this study, the presence of signs of shock was associated with patients with a higher mean age and higher mortality due to infectious diseases. On the other hand, patients who did not show signs of shock had a higher percentage of discharge and survival in the first 24 hours, six months after CRA and one year after CRA.

Patients who experience a CRA, in most cases, require critical and long-term care after the event and undergo invasive procedures, factors that make them vulnerable to local and systemic infections. A high incidence of sepsis is reported in the world according to literature^23^ ^-^ ^25^ and the increasing number of elderly and patients with chronic diseases are factors, among others, that may contribute to increased mortality in these situations^23^. A post-CRA care plan has the potential to avoid early mortality caused by hemodynamic instability and multiple organ failure, as well as late morbidity and mortality resulting from persistent neurological damage. Thus, such measures should be encouraged and disseminated^26^.

Among the limitations of this study we can highlight the fact that secondary data were used, obtained from medical records, which sometimes have incomplete information. Furthermore, although the study site was a high-complexity university hospital, it has limited resources and this may have influenced the findings.

This study is of extreme relevance to the practice because the warning signs preceding a CRA are common and may be manifested through changes in vital signs and occurrence of signs and symptoms. The periodical monitoring, according to the needs of the patients, of vital signs and provision of full and uninterrupted care are fundamental activities of the nursing team. By doing so, the nursing team is able to identify signs and symptoms preceding cardiocirculatory collapse in a timely manner.

## Conclusion

In this study, the shock signs, neurological signs, malaise, and ACS were identified as warning signs. The most prevalent VS changes were found in HR, RR and O2 saturation. Patients with severely abnormal SBP were not discharged and those with abnormal respiratory rate did not survive six months after CRA.
